# CTNNB1 Genetic Variation and Its Interaction With DLK1 in Type 2 Diabetes Mellitus

**DOI:** 10.1096/fj.202600107R

**Published:** 2026-04-10

**Authors:** Xiaoxue Gan, Yilinuer Adeerjiang, Min Xu, Xuan He, Sheng Jiang, Guoli Du

**Affiliations:** ^1^ State Key Laboratory of Pathogenesis, Prevention, and Treatment of High Incidence Diseases in Central Asia Urumqi Xinjiang People's Republic of China; ^2^ Department of Endocrinology First Affiliated Hospital of Xinjiang Medical University Urumqi Xinjiang People's Republic of China; ^3^ Department of Endocrinology Hotan Prefecture People's Hospital Hotan Xinjiang People's Republic of China; ^4^ Department of Endocrinology Bayingolin Mongolian Autonomous Prefecture People's Hospital Kuerle Xinjiang People's Republic of China

**Keywords:** CTNNB1, DLK1, gene polymorphism, type 2 diabetes mellitus, Wnt/β‐catenin pathway

## Abstract

To investigate the association between Catenin Beta 1 (CTNNB1) gene polymorphisms and type 2 diabetes mellitus (T2DM), as well as the interacting molecules of CTNNB1. Bioinformatic analysis of publicly available transcriptomic datasets derived from T2DM samples identified a panel of differentially expressed genes (DEGs), among which CTNNB1 exhibited significant differential expression. Subsequent functional enrichment analysis and molecular docking revealed a co‐expression network between CTNNB1 and Delta‐like 1 homologue (DLK1). A total of 988 subjects including 455 patients with T2DM and 533 healthy controls were included, and the epidemiological and clinical data were collected. Association between SNPs (rs2953, rs4135387, rs1798802) of CTNNB1 and susceptibility to T2DM was analyzed. CTNNB1 expression was quantified in human serum by ELISA. A mouse model of diabetes was induced by a high‐fat diet (HFD). The weight, blood glucose levels, and pancreatic morphology were detected. In a diabetes mouse model, CTNNB1 and DLK1 levels were further assessed using ELISA, Western blot, qRT–PCR, and immunohistochemistry. Immunofluorescence staining was also performed on pancreatic tissue sections to evaluate the spatial relationship within islets between β‐catenin and DLK1. Integrated analysis of DEGs and pathway enrichment identified CTNNB1 as a key gene in T2DM. Functional enrichment and molecular docking suggested that CTNNB1 and DLK1 may interact through hydrogen bonding. The CTNNB1 gene rs1798802 locus was associated with T2DM. The proportion of T2DM patients was lower in the GG genotype group than in controls (47.7% vs. 52.3%). The GG genotype of the CTNNB1 gene at the rs1798802 tended to protect the individuals from T2DM (OR = 0.640, 95% CI: 0.446–0.918, *p* = 0.015). Serum levels of CTNNB1 were significantly reduced in patients with T2DM (74.5 pg/mL vs. 23.78 pg/mL). Compared with control mice, HFD‐induced T2DM mice showed significantly decreased protein content and gene expression of β‐catenin in the pancreatic tissue, while a concurrent decrease was also observed in DLK1 expression. Immunofluorescence staining revealed a significant overlap in the subcellular distribution of CTNNB1 and DLK1, which supported their potential physical interaction within pancreatic islet cells. The CTNNB1 gene exhibits differential expression in T2DM, and its genetic polymorphisms are also associated with the disease. The CTNNB1 gene rs1798802 loci GG genotype may be a protective factor against T2DM. CTNNB1 may interact with DLK1, potentially contributing to the regulation of pancreatic islet function. The Wnt/β‐catenin signaling pathway is inhibited in pancreatic islets in T2DM. β‐catenin (CTNNB1) is a potential target for the treatment of T2DM.

## Graphical abstract

This graphical abstract illustrates the integrated study of CTNNB1 and DLK1 interaction in type 2 diabetes mellitus (T2DM). Combining bioinformatics, human genetic analysis, and HFD‐induced mouse models, it shows CTNNB1 and DLK1 synchronous downregulation in T2DM, the protective effect of CTNNB1 rs1798802 GG genotype, and cooperative colocalization of CTNNB1 and DLK1 in pancreatic islets, suggesting a potential common regulatory mechanism in T2DM pathogenesis.

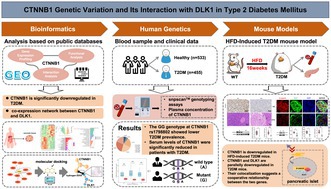

## 1 Introduction

Type 2 diabetes mellitus (T2DM) is a chronic metabolic disorder characterized by hyperglycemia. Chronic hyperglycemia is caused by impaired insulin sensitivity and β‐cells' function. The chronic metabolic disorder is influenced by a complex interplay of genetic and environmental factors, including age, sex, ethnicity, lifestyle, and obesity, contributing to its development [1, 2].

The Wnt/β‐catenin signaling pathway has been widely acknowledged to play an important role in pancreatic endocrine cell proliferation, secretion and viability [3]. The loss of functional β‐cells is a crucial event in the development of chronic hyperglycemia in T2DM [3].

β‐catenin is an integral cell–cell adhesion adaptor protein as well as a transcriptional coregulator that is encoded by the CTNNB1 gene [4]. Recent studies have indicated that activation of the Wnt/β‐catenin pathway promotes the regeneration of pancreatic β‐cells, with β‐catenin expression serving as a proliferation marker in adult murine β‐cells [5]. Accordingly, constitutive activation of β‐catenin is associated with lower basal glycemia, improved glucose tolerance, and elevated insulin levels, accompanied by an increase in β‐cells volume [3, 6]. These findings have been corroborated by several in vitro studies. In various β‐cells lines (MIN6, NIT1, and INS‐1 cells) as well as in isolated islets, treatment with synthetic Wnt3a or Wnt‐enriched medium led to activation of the Wnt/β‐catenin pathway, resulting in significant β‐cells proliferation and enhanced glucose‐stimulated insulin secretion [7, 8, 9]. Research employing high‐fat diet (HFD)‐induced prediabetes mouse models has revealed that beta‐cell proliferation occurs specifically during the prediabetic phase, accompanied by compensatory activation of the Wnt signaling pathway and upregulation of CTNNB1 gene expression [5]. In contrast, Transcription Factor 7‐Like 2 (TCF7L2), a downstream effector of the Wnt/β‐catenin signaling pathway, is critically involved in islet function, insulin signaling, and closely associated with T2DM susceptibility. A previous study investigated that demonstrated under identical HFD conditions, TCF7L2‐knockout mice exhibited a significant reduction in β‐cells mass compared to wild‐type mice [10]. To further investigate whether the activation status of the Wnt pathway and its downstream target genes varies across different stages of T2DM, relevant studies have shown that during 8 weeks of HFD feeding, mice exhibited increased β‐cells mass and elevated pancreatic TCF7L2 levels. In contrast, after 12 weeks of HFD feeding, TCF7L2 expression was downregulated, accompanied by the onset of hyperglycemia and β‐cells failure [11]. According to current research, although activation of the Wnt pathway has been shown to promote pancreatic islet cell proliferation and improve glucose homeostasis, considerable controversy remains regarding whether Wnt signaling is activated or suppressed within pancreatic tissue in the T2DM.

At the genetic level, numerous studies have indicated that the pathogenesis of T2DM is also associated with genetic polymorphisms. A study revealed that individuals carrying the rs7903146 T2DM risk allele exhibited elevated TCF7L2 expression in pancreatic islets, which correlated with impaired insulin secretion and diminished incretin responsiveness [12]. Individuals carrying the A allele (GA + AA genotypes) at the rs3765467 locus of Glucagon‐Like Peptide‐1 Receptor (GLP‐1R) exhibited significantly lower HbA1c levels compared to those with the GG genotype [13]. Whether genetic polymorphisms in CTNNB1 also influence pancreatic beta‐cell function and contribute to the pathogenesis of T2DM remains a subject of investigation.

In this study, we employed bioinformatic approaches to identify differentially expressed genes (DEGs) in T2DM, with a specific focus on evaluating the expression of CTNNB1. Enrichment analysis was further conducted to predict potential molecular interactions involving CTNNB1, followed by molecular docking simulations to characterize the binding modalities between CTNNB1 and its putative partners. Meanwhile, we sequenced single nucleotide polymorphisms (SNPs) of the CTNNB1 gene in patients, and the differences in the distribution frequencies of the SNPs were compared between T2DM patients and control individuals. The associations between CTNNB1 gene polymorphisms and T2DM were analyzed. Animal model of T2DM was subsequently established by simple high‐fat feeding to simulate the pathogenesis of T2DM in patients, the effects of changes in islet function on CTNNB1 expression were explored. The association between CTNNB1 and its interacting molecules was subsequently verified using animal models.

## 2 Materials and Methods

### 2.1 Enrichment Analysis of Target Genes

To identify DEGs in T2DM, we obtained microarray gene expression profiles derived from human pancreatic tissue through the Gene Expression Omnibus (GEO) under accession number GSE25724 (platform GPL96). The dataset comprised 6 T2DM cases and 7 non‐diabetic controls. Raw expression data from both groups were integrated and normalized using the robust multiarray average (RMA) algorithm to ensure comparability across samples [14]. Next, we used the R “limma” package for differential gene expression analysis, with |log2FC| > 1.0 and adjusted *p* < 0.05 as the criteria for statistical significance [15]. Following initial screening, a total of 50 differentially expressed genes were identified. Expression distribution plots were subsequently generated to visualize the fold changes of these genes. To further refine the list of candidate genes, a protein–protein interaction (PPI) network analysis was conducted.

### 2.2 GO and KEGG Enrichment Analyses

Functional enrichment analysis of DEGs. Gene Ontology (GO) enrichment analysis was conducted to identify predominant biological processes, molecular functions, and cellular components. Simultaneously, the Kyoto Encyclopedia of Genes and Genomes (KEGG) pathway analysis was employed to uncover key signaling and metabolic pathways. These analyses were carried out using the R “clusterProfiler” package with a significance threshold of adjusted *p* < 0.05. The false discovery rate (FDR) was controlled using the Benjamini‐Hochberg procedure. Results were visualized using enrichment maps to facilitate interpretation of large‐scale datasets.

### 2.3 Molecular Docking Verification

Based on the results of PPI network analysis, proteins associated with metabolic regulation and tissue regeneration were screened for molecular docking with the core target CTNNB1. The crystal structure of CTNNB1 was obtained from the RCSB Protein Data Bank (https://www.rcsb.org/). Protein preparation included removal of water molecules, addition of hydrogens, and energy minimization using the UCSF Chimera package. The binding site was defined based on the location of the native ligand or key residues reported in prior studies. Ligand structures were downloaded from TCMSP database and energy‐minimized using ChemOffice software. The binding affinity was evaluated by molecular docking simulations using AutoDock Vina. The predicted binding energy of the protein–protein complex was obtained, and the docking pose was visualized with PyMOL.

### 2.4 Study Population

The present study recruited participants from the inpatient department of the First Affiliated Hospital of Xinjiang Medical University between December 2022 and December 2024. The cohort consisted of 455 individuals diagnosed with T2DM (146 females and 309 males) and 533 healthy controls (243 females and 290 males). This study was approved by the Ethics Committee of the First Affiliated Hospital of Xinjiang Medical University (Approval No. K202306‐14). All procedures were performed in strict compliance with the ethical standards of the committee and the 1964 Declaration of Helsinki and its later amendments. All patients provided written consent for their samples to be collected.

Explicit inclusion and exclusion criteria were established for this study. For the T2DM group, participants were included if they met the aforementioned WHO diagnostic criteria for T2DM, were aged 18–75 years with clear consciousness to cooperate in data collection, had complete medical records, and had residual peripheral blood specimens available from routine clinical examinations for use in the study. T2DM was diagnosed according to the WHO diagnostic criteria, including fasting plasma glucose levels ≥ 7.0 mmol/L, HbA1c levels ≥ 6.5%, 2‐h oral glucose tolerance test (OGTT) blood glucose level greater than 11.1 mmol/L, and/or the prescription of antidiabetic medications [16, 17]. For the healthy control group, participants were included if they had no evidence of diabetes or impaired glucose regulation. Exclusion criteria: (1) diagnosed with other types of diabetes mellitus; (2) had severe diabetes‐related complications requiring intensive clinical intervention; (3) were pregnant or lactating; (4) suffered from acute or chronic diseases that might interfere with glucose metabolism; (5) had a history of alcohol or drug abuse within the past 6 months; (6) were taking medications known to affect glucose tolerance; or (7) were unable to cooperate with the study or had incomplete medical records.

### 2.5 General Data Collection

Patient information was retrieved from the hospital's medical records system by searching with names and hospitalization certificate numbers. Data meeting the inclusion criteria were extracted and systematically recorded in an Excel spreadsheet. The collected general information comprised sex, age, body mass index (BMI), smoking history, as well as hypertension and coronary artery disease status. Additionally, laboratory findings related to blood glucose and lipid profiles, including cholesterol, triglycerides, high‐density lipoprotein cholesterol (HDL‐c), and low‐density lipoprotein cholesterol (LDL‐c), were also gathered.

### 2.6 Genotyping Assay

Subsequently, single‐nucleotide polymorphisms (SNPs) located within CTNNB1 were retrieved from the HapMap database (www.hapmap.org), retaining only those with minor allele frequencies (MAF) exceeding 5% [18]. A 5 mL sample of fasting peripheral venous blood was collected from each participant using EDTA‐containing vacuum tubes. Following centrifugation, plasma and cellular components were separated and stored at −80°C for subsequent analysis. Plasma samples were analyzed for routine biochemical indicators, while genomic DNA was isolated from the blood cells with a commercial whole‐blood genome extraction kit (Tiangen Biotech, China). Genotyping for CTNNB1 rs2953, rs4135387, rs1798802 was conducted using SNPscan technology, with technical assistance from Center for Genetic and Genomic Analysis, Genesky Biotechnologies Inc. (Shanghai, China). A randomly selected 5% of samples were genotyped to make sure the quality and reproducibility.

### 2.7 Animal Studies

C57BL/6 mice (6 weeks old, weighing 15–20 g) were procured from the Animal Center of Xinjiang Medical University. The animals were housed under specific pathogen‐free (SPF) conditions, maintained at 22°C ± 2°C with 50% ± 15% humidity and a 12‐h light/dark cycle. Food and water were provided ad libitum throughout the study. Prior to inducing T2DM, all the mice underwent a 1‐week acclimatization period with normal chow.

Mice were randomly divided into two groups: the control group was fed a standard diet (MD17111, Medicience, Jiangsu, China), while the T2DM group was fed a high‐fat diet (HFD, 60% kcal from fat; D09100310, Beijing Huafukang Bioscience, China) for 16 weeks. Following the 16‐week dietary intervention, subsequent experiments were performed (*n* = 3–7 per group) [19]. The experimental unit was the individual animal. The body weights of the two groups of mice were monitored and recorded every 2 weeks during the modeling period. All animal experiments were reported in accordance with the ARRIVE guidelines. This study and the animal experimental protocols were reviewed and approved by the Ethics Committee of the First Affiliated Hospital of Xinjiang Medical University (Approval No. IACUC‐JT‐20230627‐30).

### 2.8 Intraperitoneal Glucose Tolerance Test (IPGTT) and Insulin Tolerance Test (ITT)

Two weeks prior to the conclusion of the modeling period, an intraperitoneal glucose tolerance test (IPGTT) was administered. Following a 16‐h fasting period, mice in each group received an intraperitoneal injection of glucose at a dose of 2 g/kg body weight. Tail vein sampling was used to test fluctuations in mouse blood glucose at 0, 30, 60, 90, and 120 min using a glucometer. Glucose tolerance was evaluated by calculating the area under the curve (AUC) for the glucose tolerance test. ITT was performed 1 week before the end of treatment. After starvation for 4 h, the mice in each group received an intraperitoneal injection of 0.5 U/kg human insulin [20]. Blood glucose monitoring was performed as previously described.

### 2.9 Enzyme‐Linked Immunosorbent Assay (ELISA)

Serum levels of CTNNB1 and DLK1 were quantified by ELISA according to the manufacturer's protocols (CTNNB1: E11307m, Mouse ELISA Kit; E08963h, Human ELISA Kit, CUSABIO, Guangzhou, China; DLK1: JL58147, Mouse ELISA Kit, JONLNBIO, Shanghai, China), using serum samples aliquoted and stored at −80°C until analysis [21]. For human serum analysis, 304 participants (166 controls and 138 T2DM patients) were randomly selected from a total of 988 individuals enrolled in the SNP genotyping cohort, and the level of CTNNB1 was detected in these human serum samples. For mouse serum samples, eyeballs were excised prior to dissection for blood collection. Briefly, mice were deeply anesthetized with isoflurane to minimize pain and stress during the procedure. Then eyeballs were carefully excised before dissection, and a sterile capillary tube was gently inserted into the orbital venous plexus of the excised eyeballs to collect blood into a sterile centrifuge tube. Following blood collection, sterile gauze was applied to the orbital fossa for 30 s to achieve hemostasis. The collected blood samples were then allowed to clot at room temperature for 30 min, followed by centrifugation at 3000 rpm for 15 min at 4°C to separate the serum [22]. Both CTNNB1 and DLK1 levels were measured in the mouse serum samples.

### 2.10 Histopathological Tests

Following a 12‐h fasting period, mice were anesthetized with 2% isoflurane and pancreatic tissues were surgically excised. A portion of the harvested tissues was immediately snap‐frozen in liquid nitrogen and stored at −80°C for subsequent analysis. The remaining specimens were fixed in 4% paraformaldehyde, followed by dehydration, clearing, paraffin embedding, and sectioning [23]. Sections were stained with hematoxylin and eosin (HE) following standard protocols and examined by light microscopy.

### 2.11 Immunohistochemistry

Pancreatic tissues were fixed in 4% formaldehyde, embedded in paraffin, and cut into slices (5 μm thick). Following deparaffinization and antigen retrieval, the tissue sections were incubated with 10% goat serum to block nonspecific binding sites. Then, the sections were incubated overnight at 4°C with β‐catenin antibodies (1:500 dilution; Abcam; ab32572, USA). Subsequently, they were incubated with secondary antibodies (1:1000 dilution, Solarbio) for 30 min. DAB chromogenic reagent was used to develop the color, and nuclei were counterstained with hematoxylin [24]. Histological images were acquired using bright‐field microscopy.

### 2.12 Western Blot

Fresh pancreatic tissue samples were lysed in 1 mL of high‐efficiency RIPA solution containing phenylmethylsulfonyl fluoride (PMSF) protease inhibitor and homogenized on ice in an ultrasonic breaker. The protein supernatant was isolated by centrifugation at 12 000 rpm for 30 min at 4°C, and its concentration was determined using a bicinchoninic acid (BCA) assay kit (Solarbio, PC0020, China). Following this, proteins was separated by 10% SDS polyacrylamide gel electrophoresis and electrotransferred to polyvinylidene fluoride (PVDF) membranes, and blocked using 5% non‐fat dry milk (BioFroxx, 1172GR500). The proteins molecular weight were identified using a Prestained Protein Marker (Servicebio, G2083‐250UL). The membranes were incubated overnight at 4°C with primary antibodies against β‐catenin (1:1000, abcam, Ab32572, USA) and DLK1 (1:1000, Huabio, HA722180), and then incubate with the second antibody (1:10 000, Solarbio). Protein bands were detected using a Bio Rad ChemiDoc MP chemiluminescence gel imaging system (California, USA) and quantitatively assessed based on their grayscale values.

### 2.13 Quantitative Real‐Time PCR (qRT‐PCR)

Total RNA was isolated from pancreatic tissue using TRIzol reagent (Thermo Fisher, USA), followed by cDNA synthesis with reverse transcription reagents (Takara #RR037A, Japan). qRT‐PCR was performed using ChamQ Universal SYBR qPCR Master Mix (Takara #RR820A, Japan) under the following conditions: 40 cycles of 95°C for 30 s (denaturation), 60°C for 34 s (annealing), and 72°C for 30 s (extension). Gene expression levels were normalized to GAPDH and analyzed using the $2^{-∆∆C_{T}}$ method.

### 2.14 Immunofluorescence Staining

Paraffin‐embedded murine pancreatic sections were dewaxed in xylene and rehydrated through graded ethanol to water. Antigen retrieval was performed in sodium citrate buffer (10 mM, pH 6.0) under heat. After washing, sections were blocked and permeabilized for 1 h at room temperature with 5% normal goat serum (Biosharp, BL210A) and 0.3% Triton X‐100 in PBS. Primary antibodies (1:500, abcam, Ab222754) diluted in blocking buffer were applied overnight at 4°C. Following PBS washes, fluorescent secondary antibodies (1:200, Bioss, bs‐0295G‐CY3) were incubated for 1 h at room temperature. Nuclei were counterstained with DAPI (Servicebio, G1012) and slides were mounted with anti‐fade medium [25]. Images were acquired using a laser‐scanning confocal microscope with sequential scanning.

### 2.15 Statistical Methods

All statistical analyses and data visualizations were carried out with SPSS 29.0 and GraphPad Prism 10.2. Results are expressed as mean ± standard error of the mean (SEM). Group comparisons were made using *t*‐test, and categorical data were assessed with the chi‐square (*χ* ^2^) test. To identify potential risk factors associated with T2DM, logistic regression analysis was employed, considering results statistically significant at *p* < 0.05.

## 3 Results

### 3.1 Identification of Differentially Expressed Genes

**FIGURE 1:**
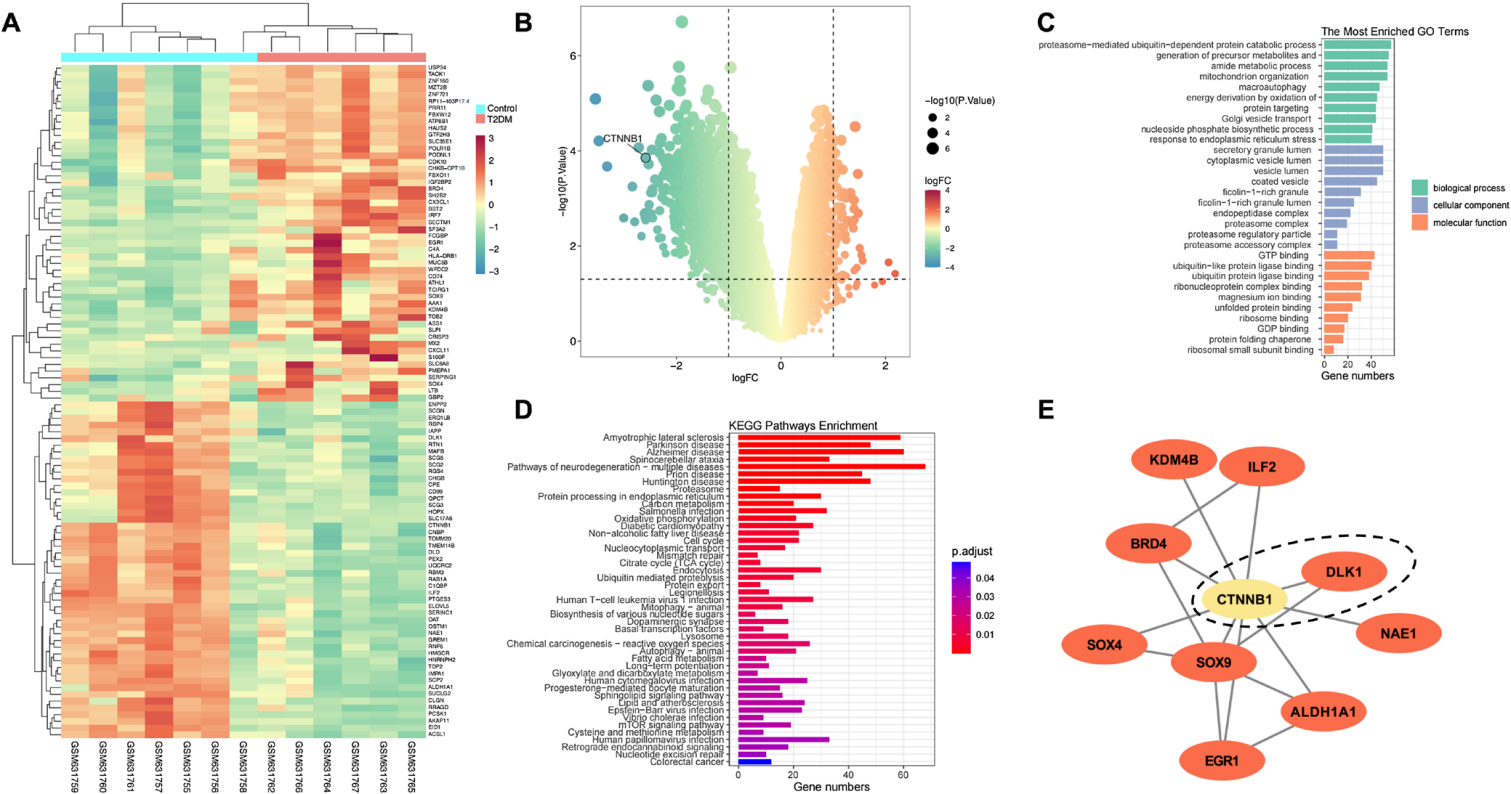
Identification of differentially expressed genes (DEGs) in type 2 diabetes mellitus (T2DM) (GSE25724) using GEO2R. (A) The heatmap depicts the expression profile of CTNNB1, showing a significant reduction in its expression levels in T2DM samples compared to controls. (B) Volcano plots of the DEGs in GSE25724. The negative log10‐transformed adjusted *p* values (*y* axis) are plotted against the average log2‐fold changes (*x* axis) in gene expression. The identified DEGs are shown in red (log2FC > 1) and green (log2FC < −1) with adjusted *p* < 0.05. CTNNB1 is labeled, confirming its significant downregulation. (C) To identify the functional categories of DEGs between healthy individuals and T2DM through GO annotation. The enriched GO terms with *p* < 0.05 included biological process (BP), cellular component (CC) and molecular function (MF). (D) KEGG pathway analysis. (E) Protein–protein interaction (PPI) network establishment and identification of hub genes. CTNNB1 interacts with DLK1 and they serve as top‐ranked hub genes and their direct protein interaction, paly critical roles in metabolic processes, underscoring their potential importance in T2DM.

In GSE25724, 13 237 variables exist, with the red dots indicating log2FC > 1 and the green dots indicating log2FC < −1. Among the 1138 DEGs, 59 genes were upregulated, and 1079 genes were downregulated. In the heatmap from unsupervised hierarchical clustering analysis, healthy individuals (*n* = 7) presented substantially different gene expression profiles from those of T2DM (*n* = 6). As shown in a heatmap and volcano plot (Figure 1A,B), CTNNB1 also showed significant differential expression, with a 0.167‐fold decrease in its expression in the T2DM group compared to the control group (*p* < 0.001). These findings reveal that the expression levels of CTNNB1 in the pancreatic tissue may be associated with the risk of T2DM.

### 3.2 GO and KEGG Pathway Enrichment and PPI Network Establishment

To determine the functional roles of the DEGs, we performed GO enrichment analysis comparing healthy controls and individuals with T2DM (Figure 1C). The significantly enriched GO terms (*p* < 0.05) spanned three major categories: biological processes (BP), cellular components (CC), and molecular functions (MF).

In the BP category, the five most significantly enriched GO terms included proteasome‐mediated ubiquitin‐dependent protein catabolic process, generation of precursor metabolites and energy, mitochondrion organization, amide metabolic process, and macroautophagy; in the CC category, the five most significantly enriched GO terms included secretory granule lumen, cytoplasmic vesicle lumen, vesicle lumen, proteasome complex, and coated vesicle. In terms of MF frequency, the five most significantly enriched GO terms included GTP binding, ubiquitin‐like protein ligase binding, ubiquitin protein ligase binding, ribonucleoprotein complex binding, and magnesium ion binding.

KEGG pathway analysis revealed significant enrichment of terms related to neurodegenerative diseases, metabolic pathways, and infection‐related processes (Figure 1D). Top enriched pathways included Amyotrophic lateral sclerosis (ALS), Parkinson disease, Alzheimer disease, and Ubiquitin‐mediated proteolysis, suggesting a central role of protein homeostasis dysregulation and mitochondrial dysfunction in the studied condition.

To explore the molecular interactions among the DEGs, PPI networks were analyzed using the STRING database. The PPI network revealed that CTNNB1 interacted with metabolism‐related proteins such as DLK1 and epigenetic regulation proteins such as Aldehyde Dehydrogenase 1 Family Member A1 (ALDH1A1), NEDD8 Activating Enzyme E1 Subunit 1 (NAE1), Bromodomain Containing 4 (BRD4), Lysine Demethylase 4B (KDM4B), SRY‐Box Transcription Factor (SOX), and Early Growth Response 1 (EGR1) (Figure 1E).

### 3.3 Patient Characteristics and Genotyping

Our study comprised 455 patients with T2DM and 433 healthy controls, from whom we collected clinical data and corresponding DNA samples. As shown in Table 1, clinical features and baseline characteristics of the participants and differences in anthropometric and biochemical measures between the T2DM group and healthy controls were evaluated.

**TABLE 1:** Clinical‐demographic characteristics of the T2DM and control subjects.

	Controls (*n* = 533)	T2DM (*n* = 455)	*p*
Age, years	43.6 ± 13.1	58.7 ± 12.7	< 0.001
Gender, *n* (%)			
Male	290 (54.4)	309 (67.9)	< 0.001
Female	243 (45.6)	146 (32.1)	
Smoker, *n* (%)	98 (19.1)	127 (28.1)	0.001
Alcohol consumption, *n* (%)	199 (38.9)	130 (28.9)	0.001
Hypertension, *n* (%)	80 (15)	320 (71)	< 0.001
CAD, *n* (%)	11 (2.1)	197 (43.3)	< 0.001
Dyslipidemia, *n* (%)	0 (0)	250 (54.9)	< 0.001
SBP (mmHg)	119 ± 16	127 ± 18	< 0.001
DBP (mmHg)	74 ± 11	77 ± 11	< 0.001
Obesity, *n* (%)	154 (28.9)	325 (71.4)	< 0.001
BMI (kg/m^2^)	23.42 ± 3.07	27.34 ± 4.13	< 0.001
HbA1c (%)	5.44 ± 0.36	7.81 ± 2.30	< 0.001
FPG (mmol/L)	4.79 ± 0.48	7.77 ± 3.26	< 0.001
TG (mmol/L)	1.28 ± 0.78	2.41 ± 2.42	< 0.001
TC (mmol/L)	4.14 ± 1.18	4.36 ± 0.84	< 0.001
HDL‐C (mmol/L)	1.25 ± 0.26	0.97 ± 0.26	< 0.001
LDL‐C (mmol/L)	2.55 ± 0.88	2.78 ± 0.65	< 0.001

*Note:* *p*‐value < 0.05 was considered significant difference.

Abbreviations: BMI, body mass index; CAD, coronary artery disease; DBP, diastolic blood pressure; FPG, fasting plasma glucose; HbA1c, hemoglobin A1C; HDL‐C, high‐density lipoprotein cholesterol; LDL‐C, low‐density lipoprotein cholesterol; Obesity, BMI ≥ 28 kg/m^2^; SBP, systolic blood pressure; T2DM, type 2 diabetes mellitus; TC, total cholesterol; TG, triglyceride.

Compared to the control group, patients in the T2DM group were significantly older (58.7 ± 12.7 vs. 43.6 ± 13.1 years, *p* < 0.001), and had a higher BMI (27.34 ± 4.13 vs. 23.42 ± 3.07 kg/m^2^, *p* < 0.001). Additionally, the T2DM group showed significantly higher levels of blood pressure (SBP 119 ± 16 vs. 127 ± 18 mmHg, *p* < 0.001, DBP 74 ± 11 vs. 77 ± 11 mmHg, *p* < 0.001), FPG (4.79 ± 0.48 vs. 7.77 ± 3.26 mmol/L, *p* < 0.001), TG (1.28 ± 0.78 vs. 2.41 ± 2.42 mmol/L, *p* < 0.001), TC (4.14 ± 1.18 vs. 4.36 ± 0.84 mmol/L, *p* < 0.001), LDL‐C (2.55 ± 0.88 vs. 2.78 ± 0.65 mmol/L, *p* < 0.001), HDL‐C levels (1.25 ± 0.26 vs. 0.97 ± 0.26 mmol/L, *p* < 0.001), and HbA1c (5.44 ± 0.36 VS 7.81 ± 2.30 mmol/L, *p* < 0.001).

### 3.4 Association Between rs1798802 Polymorphism of CTNNB1 Gene and T2DM

The CTNNB1 gene rs1798802 polymorphism was significantly associated with the risk of developing T2DM. Human carrying A allele (AA + AG genotype) at the rs1798802 locus had a greater risk of developing T2DM than those with GG genotype (50.5% vs. 40.8%, *p* = 0.003, Table 2).

**TABLE 2:** CTNNB1 gene polymorphisms in patients with T2DM and the control group.

Polymorphisms	Controls (*n* = 533)	T2DM (*n* = 455)	*p*
rs2953			
TT + TG	494 (54.2%)	418 (45.8%)	0.634
GG	39 (51.3%)	37 (48.7%)	
rs4135387			
TT + TA	504 (54.4%)	422 (45.6%)	0.292
AA	29 (46.8%)	33 (53.2%)	
rs1798802			
AA+AG	266 (49.5%)	271 (50.5%)	0.003
GG	267 (59.2%)	184 (40.8%)	

*Note:* *p*‐value < 0.05 was considered a significant difference.

Abbreviation: T2DM, type 2 diabetes mellitus.

As shown in Table 3, age, gender, BMI, TG, TC, HDL‐C, LDL‐C, and the rs1798802 GG genotype of CTNNB1 were included to construct a multivariate logistic risk model. Through univariate and multivariate logistic analysis, we found that age (OR: 1.096, 95% CI: 1.079–1.112, *p* < 0.001), BMI (OR: 1.350, 95% CI: 1.272–1.434, *p* < 0.001), and HDL‐C (OR: 0.215, 95% CI: 0.146–0.317, *p* < 0.001) may be risk factors for T2DM, while the rs1798802 GG (OR: 0.640, 95% CI: 0.446–0.918, *p* = 0.015) genotype may be a protective factor for T2DM.

**TABLE 3:** Univariate and multivariate logistic regression analyses of risk factors for T2DM.

Risk factor	Univariate logistic regression		Multivariate logistic regression	
	OR (95% CI)	*p*	OR (95% CI)	*p*
rs1798802				
AA+AG	1.0 (Reference)		1.0 (Reference)	
GG	0.676 (0.525–0.871)	0.002	0.640 (0.446–0.918)	0.015
Gender				
Male	1.0 (Reference)		1.0 (Reference)	
Female	0.564 (0.435–0.732)	< 0.001	1.072 (0.721–1.594)	0.731
Age, years	1.089 (1.076–1.102)	< 0.001	1.096 (1.079–1.112)	< 0.001
BMI (kg/m^2^)	1.379 (1.314–1.446)	< 0.001	1.350 (1.272–1.434)	< 0.001
SBP (mmHg)	1.012 (1.003–1.020)	0.006	0.994 (0.983–1.006)	0.331
DBP (mmHg)	1.015 (1.004–1.027)	0.010	1.010 (0.993–1.027)	0.267
TG (mmol/L)	1.005 (0.889–1.137)	0.934		
TC (mmol/L)	1.004 (0.945–1.067)	0.890		
HDL‐C (mmol/L)				
< 1.0	1.0 (Reference)		1.0 (Reference)	
≥ 1.0	0.147 (0.110–0.197)	< 0.001	0.215 (0.146–0.317)	< 0.001
LDL‐C (mmol/L)	1.087 (0.934–1.266)	0.282		

*Note:* *p*‐value < 0.05 was considered significant difference.

Abbreviations: CI, confidence interval; OR, odds ratio.

**FIGURE 2:**
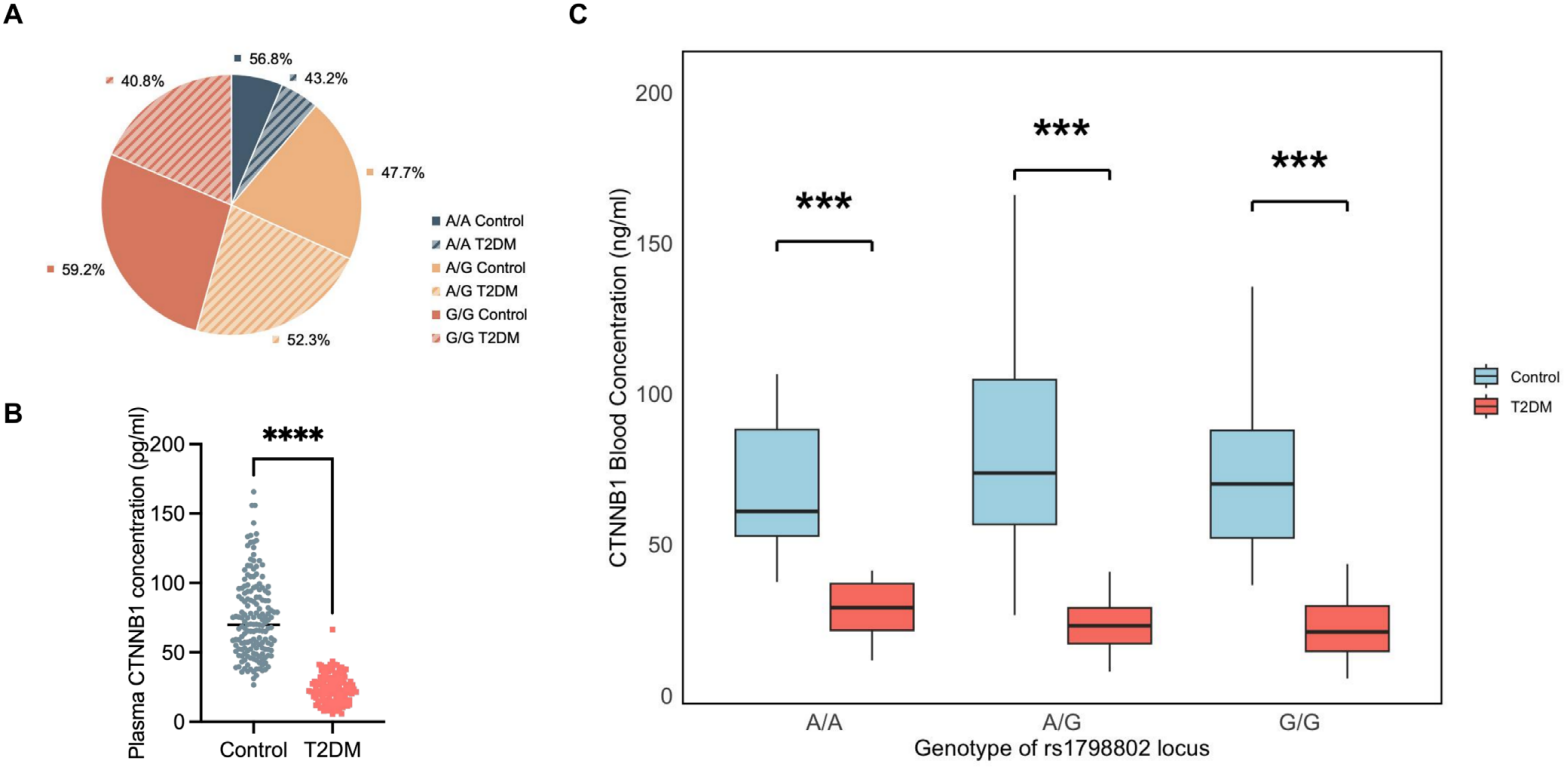
(A) The pie chart illustrates the proportional distribution of the study population across different genotypes (A/A, A/G, G/G) and their clinical status (control or T2DM). The relative proportions of control versus T2DM subjects within each genotype group are indicated. The proportion of T2DM subjects is lower than that of controls in the G/G (47.7% vs. 52.3%) genotype groups. (B) The serum levels of CTNNB1 were significantly lower in the T2DM group than in the NC group. (C) The box plot results showed that CTNNB1 gene expression level in serum was significantly decreased in T2DM regardless of the genotype. Data are presented as means ± SEM. ***p < 0.001, and ****p < 0.0001.

The genotypic distribution of the A/G polymorphism between the T2DM patients and the control subjects is presented in the pie chart (Figure 2A). The G/G genotype was most frequent in controls (59.2%) but exhibited a marked reduction in the T2DM group (40.8%). In contrast, the A/G genotype was more prevalent among T2DM patients (52.3%) than in controls (47.7%). The A/A genotype frequency was marginally lower in T2DM patients (43.2%) compared to controls (56.8%). This distinctive distribution pattern, characterized by a relative depletion of the G/G genotype in the patient cohort, suggests a potential association between this genetic locus and T2DM.

### 3.5 Decreased Serum CTNNB1 Levels in T2DM Patients

A total of 304 samples were randomly selected from the previously described study cohort used for SNP genotyping, including 166 healthy control subjects and 138 patients with T2DM. The serum levels of CTNNB1 were significantly lower in the T2DM group than in the control group (74.5 vs. 23.8 pg/mL, *p* < 0.001, Figure 2B).

The Box plot in Figure 2C showed that CTNNB1 gene expression level in serum was significantly decreased in T2DM regardless of the genotype (AA genotype: 71.0 ± 27.6 vs. 28.8 ± 9.2 ng/mL, *p* < 0.001; AG genotype: 78.8 ± 32.5 vs. 23.6 ± 8.8 ng/mL, *p* < 0.001; GG genotype: 72.5 ± 25.8 vs. 22.6 ± 11.3 ng/mL, *p* < 0.001).

### 3.6 Decreased CTNNB1 Expression in HFD‐Induced T2DM Mice

**FIGURE 3:**
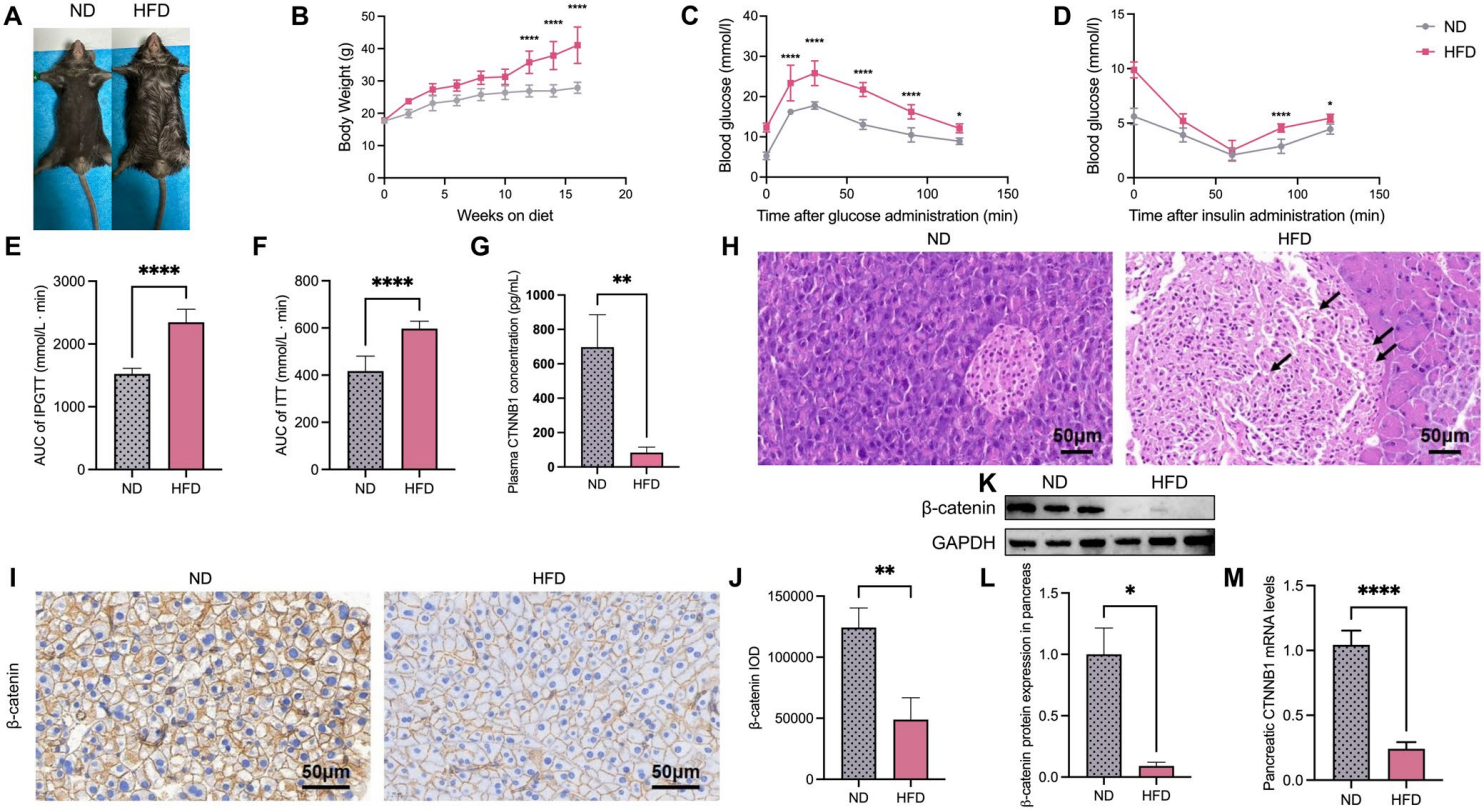
The general condition of mice after 16 weeks of high‐fat diet feeding and the decrease in CTNNB1 expression in the pancreatic tissue of diabetic mice suggest that the Wnt/β‐catenin pathway may be inhibited. (A) Representative photographs of mouse body size. (B) Body weights of HFD‐fed mice and ND‐fed mice, *n* = 7 per group. (C–F) Glucose tolerance test results and areas under the curve; insulin tolerance test results and areas under the curve (AUCs), *n* = 7 per group; (G) T2DM mice exhibited significantly lower serum levels of CTNNB1, *n* = 7 per group. (H) HE staining analysis revealed that pancreatic tissues of T2DM mice presented obvious lesions, as indicated by the unclear boundary between islet cells and exocrine glands; the increased number of blood vessels; and significant dilated congestion (black arrow), *n* = 3 per group. (I, J) immunohistochemical staining in pancreatic islets showed that β‐catenin expression was significantly inhibited in T2DM mice, *n* = 3 per group. (K–M) β‐catenin protein expression and relative mRNA levels of CTNNB1, *n* = 3 per group. Data are presented as means ± SEM. Three mice per group were randomly selected for histological, immunohistochemical, Western blot and qPCR analyses. All other data were obtained from 7 mice per group. *p < 0.05, **p < 0.01, ***p < 0.001, and ****p < 0.0001.

Subsequently, we investigated the association between T2DM and the expression profile of CTNNB1 and its downstream target DLK1 in a mouse model. T2DM mice were generated via high‐fat diet and confirmed by IPGTT (Figure 3A,B).

Compared with the control group, the HFD‐induced group presented increased food intake, decreased activity, significantly reduced self‐cleaning behavior, and increased hair oil content. The body weights of the mice in the HFD group significantly increased, and the difference was statistically significant (30.08 vs. 50.67 g, *p* < 0.05). The blood glucose level increased, as determined by a glucose tolerance test. The fasting blood glucose level surpassed 11.1 mmol/L, the blood glucose level was greater than 16.7 mmol/L 30 min after glucose injection, and the blood glucose level increased in the insulin tolerance test, indicating that the model was successfully established (Figure 3C–F) [26].

HE staining analysis revealed that pancreatic tissues of T2DM mice presented obvious lesions, as indicated by the unclear boundary between islet cells and exocrine glands; the irregular morphology, increased volume, and severe vacuolization of islets; the significantly reduced number of islet cells; the increased number of blood vessels; and significant dilated congestion (black arrow) (Figure 3H).

T2DM mice exhibited significantly lower serum levels of CTNNB1 (reduced to 12.0% of the control level, *p* = 0.005, Figure 3G). Immunohistochemical analysis showed that the expression of CTNNB1 (reduced to 39.3% of the control level, *p* = 0.006; Figure 3I,J) was significantly downregulated in the pancreatic tissues of T2DM mice compared to controls. This downregulation was further confirmed by qPCR (reduced to 23.2% of the control level, *p* < 0.001, Figure 3M) and western blot analysis (reduced to 9.1% of the control level, *p* = 0.014, Figure 3K,L).

### 3.7 DLK1 Interacts With CTNNB1 via Hydrogen Bonds and Exhibits Downregulation in T2DM Mice

**FIGURE 4:**
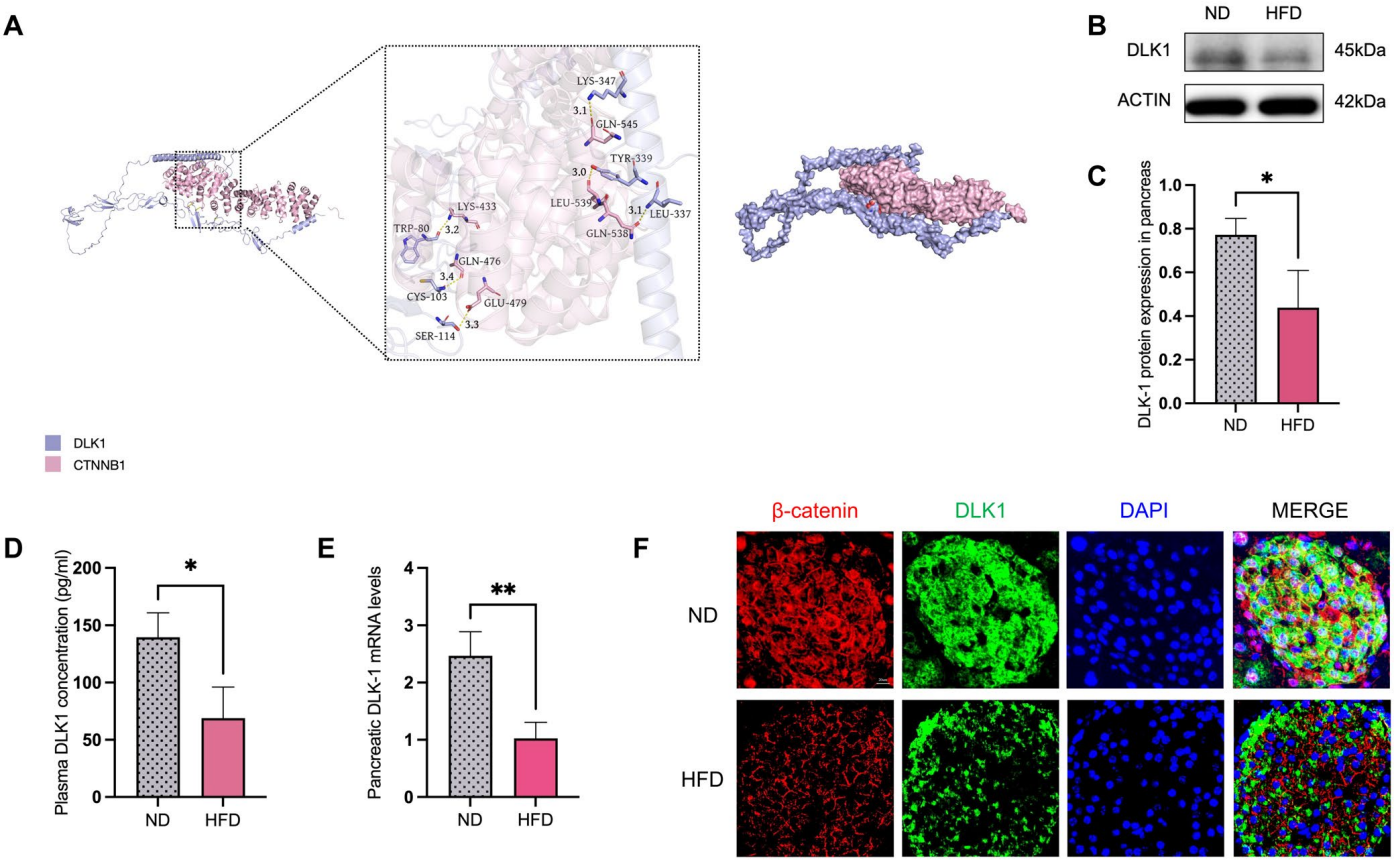
Decreased expression of DLK1 and its interaction with β‐catenin in pancreatic tissues of T2DM mice. (A) The interaction between CTNNB1 and DLK1 was assessed by molecular docking analysis. The predicted binding pose reveals a complementary interface, with critical hydrogen bonds (yellow dashed line) contributing to the binding affinity and specificity. (B, C) T2DM mice showed lower DLK1 protein expression compared with controls, *n* = 3 per group. (D) T2DM mice exhibited significantly lower serum levels of DLK1, *n* = 7 per group. (E) T2DM mice showed lower mRNA levels of DLK1, *n* = 3 per group. (F) Immunofluorescence staining of β‐catenin (red) and DLK1 (green) in pancreatic islets of control and T2DM mice. Nuclei were labeled with DAPI (blue). Compared to controls, the T2DM group showed visibly reduced signal intensity for both proteins and an attenuation of their co‐localized signals; *n* = 3 per group. Data are presented as means ± SEM. Three mice per group were randomly selected for Immunofluorescence staining, Western blot and qPCR analyses. All other data were obtained from 7 mice per group. *p < 0.05, **p < 0.01.

The interaction between CTNNB1 and DLK1 was assessed by molecular docking analysis. The calculated binding free energy of the resulting complex was −19.7 kcal/mol, suggesting a strong and specific interaction between the two proteins. This highly negative value is consistent with a spontaneous and high‐affinity binding event. Structural analysis of the top‐ranking pose revealed an extensive and complementary interface, stabilized by a network of specific intermolecular interactions, including multiple hydrogen bonds and substantial hydrophobic complementarity at the binding core. Key residues including LYS‐433, GLN‐476, GLU‐479, GLN‐545, LEU‐539, and GLN‐538 of CTNNB1 formed hydrogen bonds with TRP‐80, CYS‐103, SER‐114, LYS‐347, TYR‐339, and LEU‐337 of DLK1, respectively (Figure 4A).

We identified decreased levels of DLK1 in the serum of T2DM mice by ELISA (reduced to 49.4% of the control level, *p* = 0.001, Figure 4D). Subsequent analysis of pancreatic tissues from these diabetic mice validated the initial finding, demonstrating significant downregulation at both the protein (reduced to 56.7% of the control level, *p* = 0.03, Figure 4B,C) and RNA levels (reduced to 41.3% of the control level, *p* = 0.008, Figure 4E). Immunofluorescence analysis revealed robust and broadly distributed expression patterns for β‐catenin (red) and DLK1 (green) across pancreatic islets. Merged imaging with DAPI nuclear counterstaining (blue) showed considerable overlap between β‐catenin and DLK1 fluorescence signals, consistent with close spatial association of these proteins in islet tissue. In mice subjected to a HFD, immunofluorescence signals for both β‐catenin and DLK1 were notably diminished relative to control animals, aligning with a downregulation phenotype in the T2DM (Figure 4F).

## 4 Discussion

Bioinformatic analyses revealed decreased expression of CTNNB1 in T2DM patients. Genetic association studies further identified a correlation between CTNNB1 gene polymorphisms and susceptibility to T2DM, with individuals carrying the GG genotype at rs1798802 exhibiting a significantly lower risk of developing the disease. At the molecular level, CTNNB1 was found to interact with DLK1 through hydrogen bonding. Consistent with this finding, pancreatic tissues from diabetic mouse models showed coordinated downregulation of both CTNNB1 and DLK1, suggesting their potential synergistic involvement in the disease pathogenesis.

### 4.1 CTNNB1 Contributes to T2DM Development

The Wnt/β‐catenin signaling pathway plays a central regulatory role in pancreatic development, islet cell differentiation, and functional maintenance. Substantial evidence has demonstrated that dysregulation of this pathway is closely associated with various metabolic diseases, particularly T2DM [27]. The Wnt/β‐catenin signaling pathway is organized into four functional segments: extracellular signals, membrane components, cytoplasmic mediators, and nuclear effectors [28]. The canonical Wnt signaling pathway is primarily activated by extracellular Wnt ligands that bind to membrane receptors including Frizzled and LRP5/6 [29, 30]. As the central intracellular mediator of this pathway, β‐catenin translocates into the nucleus, where it interacts with TCF/LEF transcription factors to induce the expression of downstream target genes [31]. In the present study, the protein stability of β‐catenin was found to be tightly regulated under physiological conditions, a process frequently disrupted in T2DM. A central mechanism governing this regulation involves ubiquitin‐protein ligase binding. Under homeostatic conditions, β‐catenin undergoes continuous degradation via the ubiquitin‐dependent proteasomal pathway, maintaining its expression at low levels [32]. However, under diabetic conditions such as glucolipotoxicity (synergistic cellular toxicity induced by chronic excess glucose and free fatty acids) and oxidative stress, the activity of the β‐catenin destruction complex is often impaired. This compromises the recognition and binding of β‐catenin by ubiquitin ligases, leading to its aberrant accumulation [33, 34]. Such accumulation may drive inappropriate gene expression programs that disrupt normal β‐cells' physiology. Based on our functional enrichment analysis of CTNNB1, it exhibits multifaceted regulatory roles in β‐cells. Specifically, CTNNB1 activation positively regulates mitochondrial organization and promotes the production of precursor metabolites and energy, thereby enhancing mitochondrial biogenesis and functional capacity to meet the high energetic demands of insulin secretion. Furthermore, CTNNB1 is essential for maintaining β‐cells' identity and secretory function, directly governing the proper formation and replenishment of secretory granule lumens. Only functionally competent β‐cells can produce insulin‐rich secretory granules. Moreover, normal vesicular trafficking, relying on coated vesicles and cytoplasmic vesicle lumens, is fundamental to the insulin secretory pathway as well as to the internalization and transduction of Wnt signaling. Thus, the positive correlation between CTNNB1 and these organellar functions constitutes a compensatory adaptive mechanism in β‐cells under metabolic stress. Therapeutic strategies that target the CTNNB1 associated regulatory network to restore its physiological homeostasis, rather than merely inhibiting or activating it, may offer novel avenues for β‐cells protection and intervention in T2DM.

### 4.2 Influence of the CTNNB1 rs1798802 Variant on Type 2 Diabetes Susceptibility

In addition to bioinformatic and functional predictions, both genetic and environmental factors can influence the pathogenesis of T2DM. The correlation between genetic variation in CTNNB1 and the risk of T2DM has not been reported. In the present study, we evaluated the association between tSNPs of the CTNNB1 gene and T2DM. The results revealed that the SNP rs1798802 was significantly associated with T2DM. In addition, the rs1798802 GG genotype may be an independent protective factor for T2DM. The exact mechanism underlying this phenomenon is not yet clear; it is conceivable that its altered function could be mediated by RNA splicing, leading to aberrant β‐catenin expression [35]. A further understanding of the role of β‐catenin in pancreatic endocrine cells might reveal novel targets for the design of therapeutic strategies to counter diabetes.

### 4.3 CTNNB1‐DLK1 Interaction Modulates Pancreatic β‐Cells Function

To further clarify the regulatory network of CTNNB1 in T2DM pathogenesis, our aforementioned PPI interaction analysis revealed a potential interaction between CTNNB1 and DLK1, a metabolism‐related molecule that is closely associated with pancreatic β‐cells function and insulin secretion. As a member of the epidermal growth factor‐like superfamily, DLK1 acts as a transmembrane protein that enhances insulin biosynthesis and secretion via two distinct mechanisms [36]. One primary pathway involves DLK1‐mediated activation of the ERK signaling cascade, which stimulates the transdifferentiation of pancreatic ductal cells into β‐cells and subsequently enhances insulin production [37]. Concurrently, DLK1 enhances insulin secretion through activation of the AKT signaling pathway, which potentiates the exocytotic release of insulin from β‐cells [38, 39, 40]. DLK1 instigates a phosphorylation cascade through ERK that targets FOXO1, enabling this transcription factor to regulate PDX1 and orchestrate the nuclear translocation events necessary for islet cell development [41, 42]. A longitudinal study involving individuals with diabetes, prediabetes, and healthy controls revealed a significant inverse correlation between circulating DLK1 levels and fasting blood glucose elevation or β‐cells dysfunction. Subjects with lower baseline DLK1 levels demonstrated substantially higher cumulative incidence of diabetes compared to those with higher DLK1 concentrations [43]. Other studies suggest that DLK1 may be functionally implicated in pancreatic islet dysfunction in T2DM and could serve as a potential biomarker for predicting disease onset [39, 44]. In our study, molecular docking analysis predicted a potential binding interaction between CTNNB1 and DLK1, computationally suggesting hydrogen bonds as a likely stabilizing force. Furthermore, in vivo observations in HFD‐induced T2DM mice showed a coordinated downregulation of both CTNNB1 and DLK1 at the transcriptional and protein levels. This concurrent decrease suggests a possible functional link between the two molecules in the context of T2DM. This concurrent decrease suggests a possible functional link between the two molecules in the context of T2DM. Immunofluorescence staining provided spatial evidence supporting the interaction between CTNNB1 and DLK1 in pancreatic tissue, reinforcing their functional association in islet biology. While the Wnt/β‐catenin signaling pathway may influence pancreatic β‐cells function through DLK1, the precise regulatory mechanism requires further elucidation through subsequent functional studies.

### 4.4 Limitations and Future Perspectives

This study has several limitations. First, the bioinformatics analysis was based on a relatively small sample (6 T2DM cases and 7 non‐diabetic controls). Despite obvious differential expression patterns between groups, the small sample size and high inter‐individual variation may reduce statistical power and limit the generalizability of these preliminary results. Thus, validation in larger independent cohorts is required. Second, although we observed co‐expression of CTNNB1 and DLK1 and their potential synergistic role in T2DM, their precise regulatory mechanism remains unclear. In particular, how CTNNB1 interacts with DLK1 and modulates downstream signaling in pancreatic β‐cells needs further mechanistic exploration. Future studies should focus on functional and mechanistic experiments targeting the CTNNB1‐DLK1 interaction and its effect on Wnt/β‐catenin signaling. Elucidating their coordinated regulation of β‐cell function will not only deepen our understanding of T2DM pathogenesis, but also provide a theoretical basis and translational potential for targeted therapy, risk stratification, and personalized management of diabetes.

## 5 Conclusion

In conclusion, our study identified a critical role of CTNNB1 in the pathogenesis of T2DM and its functional synergy with DLK1. Bioinformatic analyses revealed an association between CTNNB1 and T2DM and suggested DLK1 as a potential interacting protein, while molecular docking predicted their specific binding mainly through hydrogen bonds. Human genetic analysis further demonstrated that CTNNB1 gene polymorphisms were associated with T2DM susceptibility, among which the rs1798802 GG genotype acted as a protective factor. CTNNB1 expression was significantly decreased in both human specimens and T2DM mouse models. CTNNB1 and DLK1 were coordinately downregulated and spatially colocalized in the pancreatic tissue of T2DM mice, suggesting that they may synergistically contribute to the progression of T2DM. Collectively, these findings indicate that CTNNB1 represents a potential clinically actionable target for T2DM and may regulate pancreatic β‐cells' function through DLK1.

## Author Contributions

**Sheng Jiang:** conceptualization, funding acquisition, writing – review and editing, supervision. **Guoli Du:** conceptualization, writing – review and editing, supervision, project administration. **Xiaoxue Gan:** data curation, methodology, investigation, visualization, writing – original draft. **Yilinuer Adeerjiang:** data curation, methodology, investigation, visualization. **Min Xu:** resources, methodology, investigation. **Xuan He:** data curation, formal analysis.

## Funding

The survey was funded by the Department of Science and Technology of Xinjiang Uygur Autonomous Region (Tianshan Innovation Team, 2022TSYCTD0014); Research Project on Integrated Traditional Chinese and Western Medicine for Chronic Disease Management (Project No.: CXZH2024067); the Diseases in Central Asia, Xinjiang Medical University (SKL‐HIDCA‐2024‐1, SKL‐HIDCA‐2024‐BZ5, SKL‐HIDCA‐2024‐BZ18); the Xinjiang Youth Science and Technology Top Talents Special Project (2022TSYCCX0103); the Xinjiang Bayingolin Mongolian Autonomous Prefecture “Open bidding for selecting the best candidates” project (202427) and Science and Technology Research Plan (202515). The funder had no role in the design, data collection, data analysis, and reporting of this study.

## Ethics Statement

This study was approved by the Ethics Committee of the First Affiliated Hospital of Xinjiang Medical University (Urumqi, China) and conducted according to the standards of the Declaration of Helsinki, and written informed consents were obtained from all the participants.

## Conflicts of Interest

The authors declare no conflicts of interest.

## Data Availability

The datasets used and analyzed during this study are available from the corresponding author on reasonable request.
